# Fungal cell wall agents and bacterial lipopolysaccharide in organic dust as possible risk factors for pulmonary sarcoidosis

**DOI:** 10.1186/s12995-016-0135-4

**Published:** 2016-09-21

**Authors:** Sanja Stopinšek, Alojz Ihan, Barbara Salobir, Marjeta Terčelj, Saša Simčič

**Affiliations:** 1 Institute of Microbiology and Immunology, Faculty of Medicine, University of Ljubljana, Zaloška 4, SI-1000 Ljubljana, Slovenia; 2 Department for Respiratory and Allergic Diseases, University Medical Centre, Zaloška 2, SI-1000 Ljubljana, Slovenia

**Keywords:** Sarcoidosis, Fungi, (1 → 3)-β-D-glucan, LPS, PBMC, Cytokines, Pattern-recognition receptors

## Abstract

**Background:**

Composition of organic dust is very complex, involving particles of microbial, animal and plant origin. Several environmental exposure studies associate microbial cell wall agents in organic dust with various respiratory symptoms and diseases. The aim of the present study was to investigate the in vitro effects of the co-exposure of fungal cell wall agents (FCWAs) and bacterial lipopolysaccharide (LPS) on inflammatory immune responses of peripheral blood mononuclear cells (PBMCs) from patients with pulmonary sarcoidosis.

**Methods:**

PBMCs from 22 patients with pulmonary sarcoidosis and 20 healthy subjects were isolated and stimulated in vitro with FCWAs (soluble and particulate (1 → 3)-β-D-glucan, zymosan and chitosan) and/or LPS. Subsequently, cytokines were measured by ELISA and the mRNA expression of dectin-1, toll-like receptor 2 (TLR2), TLR4 and mannose receptor (MR) was analysed by real-time RT-PCR.

**Results:**

Patients with sarcoidosis had a significantly higher secretion of inflammatory cytokines tumour necrosis factor-alpha (TNF-α), interleukin-6 (IL-6), IL-10 and IL-12 (1.7-fold, 2.0-fold, 2.2-fold, and 2.8-fold, respectively; all *p* < 0.05) after in vitro co-stimulation of PBMCs with FCWAs and LPS. We showed that PBMCs from patients with sarcoidosis had a higher baseline mRNA expression of dectin-1, TLR2, TLR4 and MR (6-fold, 11-fold, 18-fold, and 4-fold, respectively). Furthermore, we found a reduced expression of dectin-1, TLR2 and TLR4 after stimulation with FCWAs and/or LPS, although the reduction was significantly weaker in patients than in healthy subjects.

**Conclusions:**

In conclusion, co-stimulation with FCWAs and LPS of PBMC from patients with sarcoidosis caused a weaker reduction of dectin-1, TLR2, TLR4 receptors expression, which could increase the sensitivity of PBMCs, leading to excessive inflammatory cytokine responses and result in the development or progression of pulmonary sarcoidosis.

## Background

Several environmental exposure studies at workplaces or at homes associate microbial cell wall agents in organic dust with various respiratory symptoms and diseases, although, the immunopathological events involved in these diseases are very complex and not well understood. We hypothesized that exposure to fungal cell wall agents (FCWAs) and bacterial lipopolysaccharide (LPS) in organic dust may represent a risk factor for pulmonary sarcoidosis development or progression.

Sarcoidosis is a chronic granulomatous disease that most commonly affects the mediastinal lymph nodes and lungs [1]. Despite intensive research, the aetiology of sarcoidosis remains unknown. Terčelj et al. proposed a hypothesis that microbial cell wall agents, particularly agents from moulds, even in the absence of clinical infections can cause a late hypersensitivity reaction leading to granulomas [2]. In support of this hypothesis, several epidemiological studies describe the association between sarcoidosis and living in a damp and mouldy environment [3–8]. Furthermore, in clinical studies in which sarcoidosis was treated with antifungals, greater clinical improvement was reported compared with corticosteroid treatment [9, 10].

Composition of organic dust is very complex, involving particles of microbial, animal and plant origin. In mice models organic dust exposures induced the development of lymphoid aggregates, peribronchiolar or vascular inflammation, comprised of T and B lymphocytes and macrophages with associated neutrophil recruitment [11]. It has been shown that exposure to high levels of fungi and their components present in organic dust represents a risk factor for developing various respiratory symptoms and diseases, such as asthma, hypersensitivity pneumonitis, sick building syndrome and organic dust toxic syndrome [12–14]. Furthermore, bioaerosols with fungi are known to be associated with granulomatous diseases [4, 7, 15].

Bacterial LPS is one of the prime constituent in organic dust. Inhalation studies showed that LPS can cause cough, dyspnoea, nose and throat irritation, mild fever, flu-like symptoms, acute air flow obstruction, airway inflammation or asthma [16]. Furthermore, adverse effects from LPS may also be increased by other dampness-associated agents [17].

In our previous study we focused on the in vitro and in vivo effects of FCWAs in sarcoidosis. The induced in vitro secretion of cytokines from human peripheral blood mononuclear cells (PBMCs) was higher from subjects with sarcoidosis than from controls. A significant relationship was observed between disease severity, measured as chest X-ray scores indicating granuloma infiltration, and the particulate (1 → 3)-β-D-glucan-induced secretion of cytokines [18–20]. The aim of the present study was to investigate the in vitro effects of the co-exposure of FCWAs and LPS on inflammatory immune responses of PBMCs from patients with sarcoidosis. We evaluated the FCWAs influence on the in vitro cellular cytokine response to an inflammatory challenge with LPS in sarcoidosis and on the mRNA expression of the main pattern-recognition receptors (PRRs) for recognizing FCWAs and LPS, dectin-1, toll-like receptor 2 (TLR2), TLR4 and mannose receptor (MR).

## Methods

### Subjects

The study group consisted of 22 patients newly diagnosed with pulmonary sarcoidosis stage II and III according to the established criteria by the American Thoracic Society (ATS), European Respiratory Society (ERS) and World Association of Sarcoidosis and Other Granulomatous Disorders (WASOG) [21], recruited at the Department for Respiratory and Allergic Diseases, University Medical Centre Ljubljana, Slovenia in the period from September 2008 to January 2012. Patient characteristics are shown in Table 1. The exclusion criteria were pulmonary sarcoidosis stage I and IV, CD4/CD8 ratio in the bronchoalveolar lavage (BAL) less than 4, smoking and receiving any immunosuppressive therapy.

**Table 1:** Patient and healthy subject characteristics

Characteristic	Sarcoidosis patients	Healthy subjects
N	22	20
Gender		
female	12	13
male	10	7
Age in years mean (range)	44.7 (28–57)	39.9 (25–52)
Smoking	No	No
CD4/CD8 ratio in BAL mean (SD)	7.5 (3.0)	
Chest X-ray		
Stage I	0	
Stage II	15	
Stage III	7	
Stage IV	0	
Therapy	None	None

The control group consisted of 20 healthy blood-donor volunteers without any respiratory symptoms or diseases, autoimmune diseases or acute infections. The study was approved by the National Medical Ethics Committee of the Republic of Slovenia (number 122/11/08) and written informed consent was obtained from all the participants.

### Reagents and preparation of FCWAs and LPS

All reagents were commercially obtained from Sigma-Aldrich Corp. (USA), unless otherwise stated. Reagents and FCWAs were endotoxin-free and were prepared exactly as previously described [18]. Briefly, the soluble and the particulate forms of (1 → 3)-β-D-glucan (BGS and BGP) consisted of curdlan from *Alcaligenes faecalis* var. *myxogenes* (Wako Pure Chemical Industries, Japan). BGS was prepared from a suspension of curdlan powder in 0.3 M sodium hydroxide (NaOH) heated at 80 °C in a water bath until completely dissolved. The pH of BGS was neutralized with 0.3 M hydrochloric acid before being added to cell cultures. Curdlan resuspended in RPMI-1640 medium supplemented with 25 mM Hepes buffer (RPMI-1640 medium) represented BGP. Zymosan A from *S. cerevisiae* (ZYM) was prepared from a suspension of ZYM powder, boiled in 0.25 M NaOH and resuspended as a 7.5 mg/ml stock solution in RPMI 1640 medium. A low molecular weight (Mw 50–190 kDa) chitosan from crab shells (75–85 % deacetylated chitosan, CHT) was prepared as a 2 mg/ml stock solution in 0.2 % acetic acid. The final concentration in the cell cultures of all FCWAs was 200 μg/ml. LPS from *Escherichia coli* (strain 0111:B4) was dissolved as a 1 mg/ml stock solution in water and further diluted in a cell culture medium to the final concentration of 10 ng/ml.

### Isolation and stimulation of PBMCs

The model that we previously described was used for in vitro stimulation of PBMCs [18]. Briefly, PBMCs from patients with sarcoidosis and healthy subjects were isolated from freshly drawn venous blood with EDTA by density gradient centrifugation with Ficoll-Paque™ (GE Healthcare, UK). The cells were cultured in RPMI-1640 medium supplemented with 100 U/ml penicillin, 100 μg/ml streptomycin, 2 mM L-glutamine and 10 % heat-inactivated human serum (Sigma-Aldrich Corp., USA). The 1 × 10^6^ cells (final culture volume of 1.5 ml) were seeded in 24-well culture plates (Corning Costar, USA) with medium alone, with LPS (10 ng/ml), with FCWAs, or with LPS and FCWAs at 37 °C in a humidified atmosphere of 5 % CO_2_ in air. The cell-free supernatants were collected after 4 and 18 h of incubation and stored at −30 °C before further analysis.

For mRNA expression studies, 1.2 × 10^5^ PBMCs (final culture volume of 180 μl) were plated in 96-well culture plates (Greiner Bio-One GmbH, Germany) in medium alone or with FCWAs (200 μg/ml) in the absence or presence of LPS (10 ng/ml) at 37 °C in a humidified atmosphere of 5 % CO_2_ in air for 4 h.

### Real-time reverse transcription polymerase chain reaction (RT-PCR)

The mRNA expression studies were performed as previously described [18]. Briefly, the total cellular RNA was extracted on an ABI Prism 6100 Nucleic Acid PrepStation (Applied Biosystems, Foster City, USA) according to the manufacturer’s instructions. RNA was eluted in 150 μl of elution solution and stored at −80 °C until required. Ten microliters of total RNA was reverse transcribed in a 27 μl reaction mixture with a High Capacity cDNA Reverse Transcription kit (Applied Biosystems) according to the manufacturer’s instructions on an ABI GeneAmp PCR System. Real-time PCR was performed on an ABI StepOnePlus Realtime PCR instrument using a TaqMan® Universal PCR Master Mix with predeveloped TaqMan Gene Expression Assay primers and probes (Dectin-1 Hs00224028_m1, TLR2 Hs00610101_m1, TLR4 Hs01060206_m1 and MR Hs00267207_m1), according to the manufacturer’s instructions (Applied Biosystems). The internal endogenous control used was 18S rRNA. Quantification was performed with the comparative 2^−ΔΔCt^ method [22]. The amount of target gene was normalized to the internal control gene (18S rRNA) and the relative expression of target genes in cultured PBMCs was calculated in relation to the mean values of target gene expression in healthy subjects after 4 h of incubation in medium alone.

### Cytokine measurements

Cytokine concentrations in cell culture supernatants were measured by commercially available enzyme-linked immunosorbent assay (ELISA) kits. Tumour necrosis factor-alpha (TNF-α) (Milenia Biotec, Germany) was measured after 4 h of incubation. The concentrations of interleukin-6 (IL-6), IL-10 and IL-12 (Thermo Scientific, USA) were measured after 18 h of incubation.

### Statistical analysis

All statistical analyses were performed using PSAW/SPSS for Windows version 18 (SPSS Inc., IBM Company, USA). Results are presented as the mean +/− standard error of the mean (SEM). Statistically significant differences in cytokine concentration or in mRNA gene expression between the two groups of subjects were estimated by the nonparametric Mann-Whitney test. *P* values less than 0.05 were considered statistically significant.

## Results

### In vitro inflammatory cytokine response to FCWAs and LPS by PBMCs from patients with sarcoidosis

**Fig. 1:**
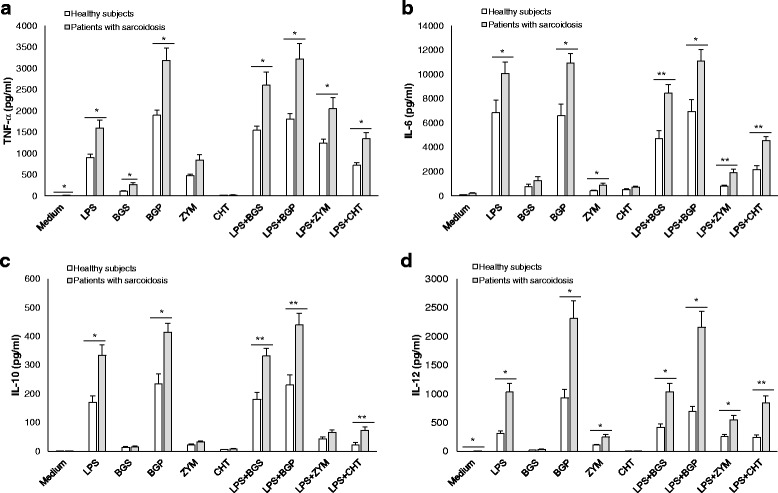
Cytokine responses of PBMCs after in vitro stimulation with FCWAs and/or LPS. PBMCs (1 × 10^6^ cells/ml) were isolated from venous blood of patients with sarcoidosis (*n* = 22) and healthy subjects (*n* = 20) and incubated with medium alone or stimulated with FCWAs (200 μg/ml) and/or LPS (10 ng/ml). Supernatant TNF-α (pg/ml) (**a**) after 4 h of incubation, IL-6 (pg/ml) (**b**), IL-10 (pg/ml) (**c**) and IL-12 (pg/ml) (**d**) after 18 h of incubation were measured by ELISA. The results are presented as the mean cytokine concentration in culture supernatants with SEM. *p < 0.05, **p < 0.001: significantly different cytokine production between patients with sarcoidosis and healthy subjects. LPS: lipopolysaccharide, BGS: soluble (1 → 3)-β D-glucan, BGP: particulate (1 → 3)-β-D-glucan, ZYM: zymosan, and CHT: chitosan

After co-stimulation of PBMCs with FCWAs and LPS, the production of TNF-α, IL-6, IL-10 and IL-12 was significantly higher in patients with sarcoidosis, compared to healthy subjects (1.7-fold, 2.0-fold, 2.2-fold, and 2.8-fold, respectively; all *p* < 0.05) (Fig. 1). Fold changes are mean values calculated from all four combinations of FCWAs and LPS. Patients with sarcoidosis thus elicited higher levels of in vitro inflammatory cytokine response of PBMCs after co-stimulation with FCWAs and LPS than healthy subjects.

### PBMCs mRNA expression of dectin-1, TLR2, TLR4, and MR at baseline and after in vitro stimulation with FCWAs and/or LPS in patients with sarcoidosis

**Fig. 2:**
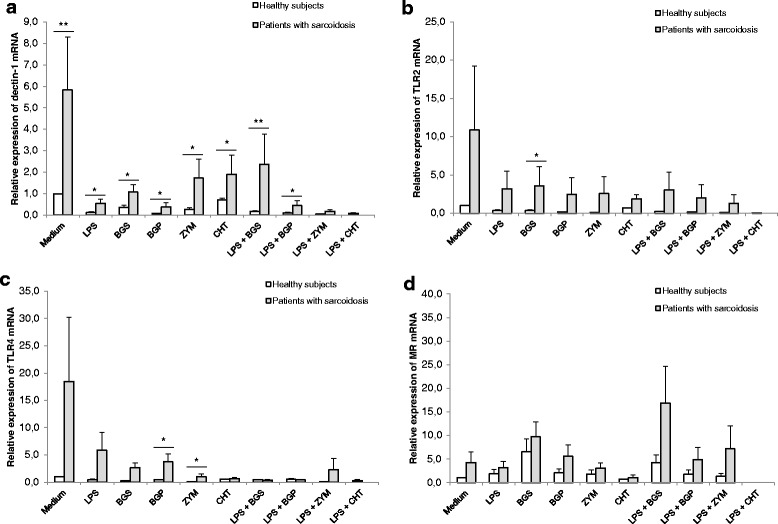
Dectin-1, TLR2, TLR4 and MR mRNA expression in PBMCs. PBMCs (1.2 × 10^5^) from sarcoidosis patients (*n* = 5) and healthy subjects (*n* = 5) were incubated in vitro with medium alone or stimulated with FCWAs (200 μg/ml) and/or LPS (10 ng/ml). After 4 h of incubation the mRNA levels of dectin-1 (**a**), TLR2 (**b**), TLR4 (**c**) and MR (**d**) were analysed by real-time RT-PCR. The results were normalized against the mRNA expression of 18S rRNA. Data are means ± SEM. *p < 0.05, **p < 0.01: significant difference of mRNA gene expression between sarcoidosis patients and healthy subjects. LPS: lipopolysaccharide, BGS: soluble (1 → 3)-β-D-glucan, BGP: particulate (1 → 3)-β-D-glucan, ZYM: zymosan, and CHT: chitosan

As shown in Fig. 2, baseline dectin-1, TLR2, TLR4 and MR mRNA expression in PBMCs was higher in patients with sarcoidosis than in healthy subjects (6-fold, 11-fold, 18-fold, and 4-fold, respectively).

When PBMCs were stimulated with FCWAs and/or LPS the mRNA expression of dectin-1, TLR2 and TLR4 was lower than at baseline in both groups of subjects. However, the expression was significantly higher in patients compared to healthy, indicating a weaker reduction of PRRs expression upon FCWAs and/or LPS stimulation in patients with sarcoidosis. Similar results were obtained after 4 and 18 h of incubation (data not shown).

## Discussion

This study was designed to investigate the in vitro effects of the co-exposure of FCWAs and LPS on inflammatory immune responses of PBMCs from patients with sarcoidosis. The main results obtained from this study show that patients with sarcoidosis had a significantly higher secretion of inflammatory cytokines TNF-α, IL-6, IL-10 and IL-12 after in vitro co-stimulation of PBMCs with FCWAs and LPS. We showed that PBMCs from patients with sarcoidosis had a higher mRNA expression of dectin-1, TLR2, TLR4 and MR at baseline. Furthermore, we found a reduced expression of dectin-1, TLR2 and TLR4 after stimulation with FCWAs and/or LPS, although the reduction was significantly weaker in patients than in healthy subjects.

The organic dust we breathe contains particles of animal, plant and microbial origin, of which the most important in relation to respiratory diseases are fungal (1 → 3)**-**β**-**D**-**glucan and bacterial LPS [14]. We demonstrated previously that after in vitro stimulation of PBMCs with FCWAs alone, the secretion of TNF-α, IL-6, IL-10 and IL-12 was higher in patients with sarcoidosis compared to healthy subjects [20]. These cytokines have all been implicated in the immunopathogenesis of sarcoidosis [1, 23–25]. Since FCWAs and LPS might have a synergistic effect on the immune responses of patients with sarcoidosis, we investigated in the present study the in vitro synthesis of inflammatory cytokines in PBMCs after co-stimulation with FCWAs and LPS. Our results showed that patients with sarcoidosis, compared to healthy subjects, had a significantly higher secretion of inflammatory cytokines TNF-α, IL-6, IL-10 and IL-12, after co-stimulation of PBMCs with FCWAs and LPS. However, it should be noted that patients with sarcoidosis included in this study had an ongoing inflammatory disease, which may have influenced the immune responses in our in vitro experiments.

In addition to having a strong impact on in vitro human PBMCs inflammatory cytokine responses, FCWAs have been shown to have various biological and immunopharmacological properties (reviewed in [26, 27]). A causal role in the development of respiratory symptoms and diseases associated with fungal exposure has been attributed to β-glucan [12, 28, 29]. β-glucan has also been shown to trigger rheumatoid arthritis in genetically susceptible mice, suggesting that fungal infection may evoke autoimmune conditions in genetically susceptible individuals [30]. Chitin has also been implicated in asthma and allergy [31]. Furthermore, it has been demonstrated that fungi [4, 32, 33] or (1 → 3)-β-D-glucan itself [34, 35] can trigger granuloma formation. In view of all this, we speculated that FCWAs in combination with LPS may evoke an exaggerated inflammatory reaction in genetically susceptible individuals and lead to sarcoid granuloma formation or progression of the disease.

Recent genetic studies suggest that PRRs might be involved in the pathogenesis of sarcoidosis [36–43]. The central PRRs involved in the recognition of fungi are C-type lectin receptors, such as dectin-1 and MR; TLRs, such as TLR-2, −4 and −9; and the galectin family proteins [44]. The key receptor involved in bacterial LPS recognition is TLR4 [45]. All biological activities of (1 → 3)-β-D-glucan are mediated by dectin-1, which also collaborates with TLR2 and TLR4. On the other hand, the immunomodulating effects of chitin and its derivatives are mediated by pathways that involve TLR2, dectin-1 and MR [46]. In our previous study, we examined the in vitro effects of FCWAs alone or in combination with LPS on PRRs mRNA expression in healthy subjects [18]. Since these receptors are suspected of being involved in the pathogenesis of sarcoidosis, we examined the effects of FCWAs and/or LPS on the mRNA expression of these receptors in PBMCs from patients with sarcoidosis.

Our results demonstrated that PBMCs from patients with sarcoidosis had a higher in vitro baseline mRNA expression of dectin-1, TLR2, TLR4 and MR than healthy subjects. The results are in accordance with Wiken et al. [37], who found that TLR2 and TLR4 expression on peripheral blood monocytes at baseline was significantly higher in patients with sarcoidosis than in healthy subjects, as measured by flow cytometry. We found a reduced expression of dectin-1, TLR2 and TLR4 after stimulation with FCWAs and/or LPS, although the reduction was significantly weaker in patients than in healthy subjects. That may indicate a defect in down-regulation of PRRs in sarcoidosis patients when exposed to FCWAs and/or LPS.

## Conclusions

In conclusion, microbial cell wall agents are airborne and poorly degradable antigens to which we are constantly exposed. Our study demonstrated that co-stimulation with FCWAs and LPS of PBMC from patients with sarcoidosis caused a weaker reduction of dectin-1, TLR2, TLR4 receptors expression, which could increase the sensitivity of PBMCs, leading to excessive inflammatory cytokine responses and result in the development or progression of pulmonary sarcoidosis.

## Abbreviations

BGP: Particulate (1 → 3)-β-D-glucan
BGS: Soluble (1 → 3)-β-D-glucan
CHT: Chitosan
FCWAs: Fungal cell wall agents
IL: Interleukin
LPS: Lipopolysaccharide
MR: Mannose receptor
PBMCs: Peripheral blood mononuclear cells
PRRs: Pattern-recognition receptors
TLR: Toll-like receptor
TNF-α: Tumour necrosis factor-alpha
ZYM: Zymosan A
